# Correction: The Hausa Back Beliefs Questionnaire: Translation, cross-cultural adaptation and psychometric assessment in mixed urban and rural Nigerian populations with chronic low back pain

**DOI:** 10.1371/journal.pone.0253681

**Published:** 2021-06-17

**Authors:** Aminu Alhassan Ibrahim, Mukadas Oyeniran Akindele, Sokunbi Oluwaleke Ganiyu, Bashir Kaka, Bashir Bello

In the External construct validity subsection of the Results, there is an error in the first sentence of the second paragraph. The cited table is incorrect. The correct sentence is: Known-groups comparison showed that the Hausa-BBQ did not significantly discriminate between patients with different age groups (p > 0.05) (Table 4).

In the Test re-test reliability subsection of the Results, there is an error in the third sentence. The cited table is incorrect. The correct sentence is: As for the subgroup analyses, the ICC, SEM, and MDC_95_ calculated for the urban (n = 42) and rural (n = 58) populations were comparable to the overall population (Table 5).
