# Supplementary material for: The Hausa Back Beliefs Questionnaire: Translation, cross-cultural adaptation and psychometric assessment in mixed urban and rural Nigerian populations with chronic low back pain
Source: PLoS One. 2021 Apr 13;16(4):e0249370. doi: 10.1371/journal.pone.0249370 (PMC8043379; doi:10.1371/journal.pone.0249370)
Supplement: S1 Appendix — (PDF) [file pone.0249370.s001.pdf]

TAMBAYOYIN BINCIKE GAME DA YARDA DA CIWON BAYA

(HAUSA VERSION OF THE BACK BELIEFS QUESTIONNAIRE)

Muna ƙoƙarin gano yadda mutane suke tunani game da matsalar ciwon baya. Ana roƙon ka bayar da ra'ayinka a faɗaɗe game da matsalar ciwon baya.

Ana roƙon ka da ka bayar da dukkan amsoshin sannan ka nuna *yardarka ko ƙin yarda* da kowane bayani ta hanyar zagaye lamba da tafi dacewa da amsar ka.

1	2	3	4	5
**SAM BAN YARDA BA**	**BAN YARDA BA**	**BANI DA ZAƁI**	**NA YARDA**	**NA YARDA SOSAI**

		**Sam ban yarda ba**				**Na yarda sosai**
1.	Babu wata tabbatacciyar hanyar samun warakar matsalar ciwon baya	1	2	3	4	5
2.	Matsalar ciwon baya zai hana ka gudanar da ayyuka a can gaba	1	2	3	4	5
3.	Matsalar ciwon baya na nufin tsayin lokacin da mutum ke fama da ciwon har ƙarshen rayuwarsa.	1	2	3	4	5
4.	Babu abin da likitoci za su iya yi game da matsalar ciwon baya	1	2	3	4	5
5.	Ciwon baya yana buƙatar a riƙa motsa jiki.	1	2	3	4	5
6.	Matsalar ciwon baya kan taɓarɓara komai na rayuwa	1	2	3	4	5
7.	Yin tiyata ne sahihiyar hanyar magance matsalar ciwon baya	1	2	3	4	5
8.	Matsalar ciwon baya na iya kasancewa ka ƙarke ana turaka a kujerar asibiti ta marasa lafiya.	1	2	3	4	5
9.	Bin hanyoyin samun waraka mafi dacewa su ne mafita ga matsalar ciwon baya	1	2	3	4	5
10.	Matsalar ciwon baya na nufin ɗaukar tsawon lokaci ba a yin aiki	1	2	3	4	5
11.	Hanya mafi sauƙi ta samun sauƙin ciwon baya ita ce shan magunguna	1	2	3	4	5
12.	Da zarar ka kamu da matsalar ciwon baya to ka kan kasance koyaushe cikin rashin kuzari	1	2	3	4	5
13.	Dole ne mai ciwon baya ya riƙa samun hutu	1	2	3	4	5
14.	Matsalar ciwon baya kan cigaba da ta'azzara idan rayuwa ta yi nisa.	1	2	3	4	5
