# Supplementary material for: The Hausa Back Beliefs Questionnaire: Translation, cross-cultural adaptation and psychometric assessment in mixed urban and rural Nigerian populations with chronic low back pain
Source: PLoS One. 2021 Apr 13;16(4):e0249370. doi: 10.1371/journal.pone.0249370 (PMC8043379; doi:10.1371/journal.pone.0249370)
Supplement: Attachment — Submitted filename: Response to Reviewers-Hausa-BBQ-R2.docx [file pone.0249370.s005.docx]

**Response letter to the reviewer's comments for the manuscript (PONE-D-20-31974R1) submitted to PLOS ONE**

**Response to Reviewer #1**

Dear reviewer,

Once again, we would like to thank you for the effort and time you spent reviewing our manuscript.

Sincerely.

**Response to Reviewer #2**

Dear reviewer,

We would like to thank you for the effort and time you spent reviewing our manuscript for the second time.

1. We have now added the response rate of the questionnaire according to the four hospitals (SEE General aspect, ceiling and floor effects, in the results section) as you suggested.
2. The limitation regarding interviewer-administration method was added in the discussion section as suggested.

You can find all the modifications/changes [text highlighted in **red** (deletions) and **purple** (additions/corrections)] in the revised file (marked version).

Sincerely.

**Response to Reviewer #3**

Dear reviewer,

We appreciate the effort and time you spent reviewing our manuscript for the second time.

You can find all the modifications/changes [text highlighted in **red** (deletions) and **purple** (additions/corrections)] in the revised file (marked version).

Please find below our response to your constructive comments/suggestions

**Comment 1:** Once again, the lines are not numbered and it is difficult to localize the comments

**Response/amendments:** We apologize for this. The numbering is supposed to be handled by the journal electronic submission process so that reviewers may be able to see the lines numbered for easy reference. However, we have added the numbering in the revised files.

**Comment 2:** Translation and cross-cultural adaptation section: I suggest to give the name of the "translators" (initials if authors or full names)

**Response/amendments:** We have now provided the initials of the forward and backward translators.

**Comment 3:** Translation and cross-cultural adaptation section, last sentence: The authors claim having sent the final version to the original developers and what happened?

**Response/amendments:** The final version of the Hausa-BBQ along with the report of the translation process was sent to the original developers for appraisal. The developers responded and there was no need for further modifications. We have now indicated this in the manuscript (SEE item 6: Proofreading).

**Comment 4:** I would suggest to replace "sample size estimation" by "population" and move the two last sentences (patients needed for reliability and validity before statistical analysis) in this section

**Response/amendments:** We replaced “Sample size estimation” with “Population” as you suggested.

**Comment 5:** In the result section, does not need to report the results in the text and in the table (external validity for example) please choose one or the other.

**Response/amendments:** The modifications were done. Table 5 was deleted and the order of the tables was reflected.

**Comment 6:** Discussion, first paragraph: for Item 4 a ceiling effect would likely mean that patients believe that doctors are unable to help them. Please check

**Response/amendments:** The sentence was checked but ceiling effects means higher scores (disagree, as the scores are reversed) and this indicate that most patients had higher scores for item 4 (Doctors cannot do anything for back trouble), [mean score (SD) = 3.06 (1.36) as shown in Table 2]. Since the scores are reversed, the interpretation will be that most of the participants disagree or do not believe that doctors cannot do anything for back trouble.

**Comment 7:** The discussion is interesting.

**Response:** Once again**,** we thank you for all your constructive suggestions and comments.

Sincerely.
